# A Case of Brunner’s Gland Hyperplasia Accompanied by an Increase in Endocrine Cells and Endocrine Cell Micronests

**DOI:** 10.7759/cureus.68688

**Published:** 2024-09-05

**Authors:** Shiori Meguro, Hideya Kawasaki, Isao Kosugi, Yasunori Enomoto, Satoshi Osawa, Ken Sugimoto, Satoshi Baba, Toshihide Iwashita

**Affiliations:** 1 Department of Regenerative and Infectious Pathology, Hamamatsu University School of Medicine, Hamamatsu, JPN; 2 Department of Preeminent Bioimaging Research, Hamamatsu University School of Medicine, Hamamatsu, JPN; 3 Department of Endoscopic and Photodynamic Medicine, Hamamatsu University Hospital, Hamamatsu, JPN; 4 First Department of Medicine, Hamamatsu University School of Medicine, Hamamatsu, JPN; 5 Department of Diagnostic Pathology, Hamamatsu University Hospital, Hamamatsu, JPN

**Keywords:** myofibroblasts, smooth muscle cells, hypergastrinemia, endocrine cell micronests, brunner's gland hyperplasia

## Abstract

Endocrine cell micronests (ECMs) are aggregates of endocrine cells known as enterochromaffin-like cells, typically measuring approximately 50 μm and usually observed in the mucosal layer of atrophic gastric fundic glands associated with hypergastrinemia. Although there are numerous reports on gastric ECMs, reports on duodenal ECMs are exceedingly rare. We report a rare case of Brunner's gland hyperplasia with increased endocrine cells and ECMs. An approximately 40 mm polyp was found in the duodenal bulb of a 57-year-old Japanese male patient during an upper gastrointestinal endoscopy, and a polypectomy was performed. Microscopic examination revealed hyperplasia of Brunner's glands in the duodenal polyp. Compared to normal Brunner's glands, hyperplastic Brunner's glands exhibited more endocrine cells. Additionally, many ECMs were observed in the fibromuscular connective tissue, comprising smooth muscle cells and myofibroblasts, adjacent to the hyperplastic Brunner's glands. The patient presented with hypergastrinemia (2,500 pg/mL; normal range: 30-140 pg/mL), and the ECMs were considered related to this condition. This case represents the first instance of a benign duodenal lesion with an increase in endocrine cells and the presence of ECMs.

## Introduction

Endocrine cell micronests (ECMs) are aggregates of enterochromaffin-like cells, typically approximately 50 μm in size, exhibiting ovoid to round or elongated morphology. ECMs are usually observed in the mucosal layer of the atrophic gastric fundic gland area associated with hypergastrinemia, such as in autoimmune gastritis, and are considered precursors to gastric carcinoids; ECMs around gastric carcinoids have also been reported [1]. Cases in which ECMs and adenocarcinoma coexist against a background of hypergastrinemia are rare but have been reported [2,3].

However, studies on ECMs in the duodenum are limited, with only four cases reported in the English literature. One report described ECMs in the duodenal bulb in a case of multiple duodenal carcinoids [4], whereas the other three studies found ECMs in the normal descending duodenum; one study reported ECMs in the normal major and minor duodenal papillae in 69 of 195 cases [5], one study found ECMs in the normal minor duodenal papilla [6], and one study reported ECMs in one of 615 consecutive duodenal specimens [7].

In this paper, we report a case of Brunner’s gland hyperplasia in the duodenal bulb with increased endocrine cells and ECMs. Additionally, we investigated the types of mesenchymal cells surrounding the ECM. To the best of our knowledge, this report is the first description of a benign duodenal lesion with ECMs.

## Case presentation

**Figure 1:**
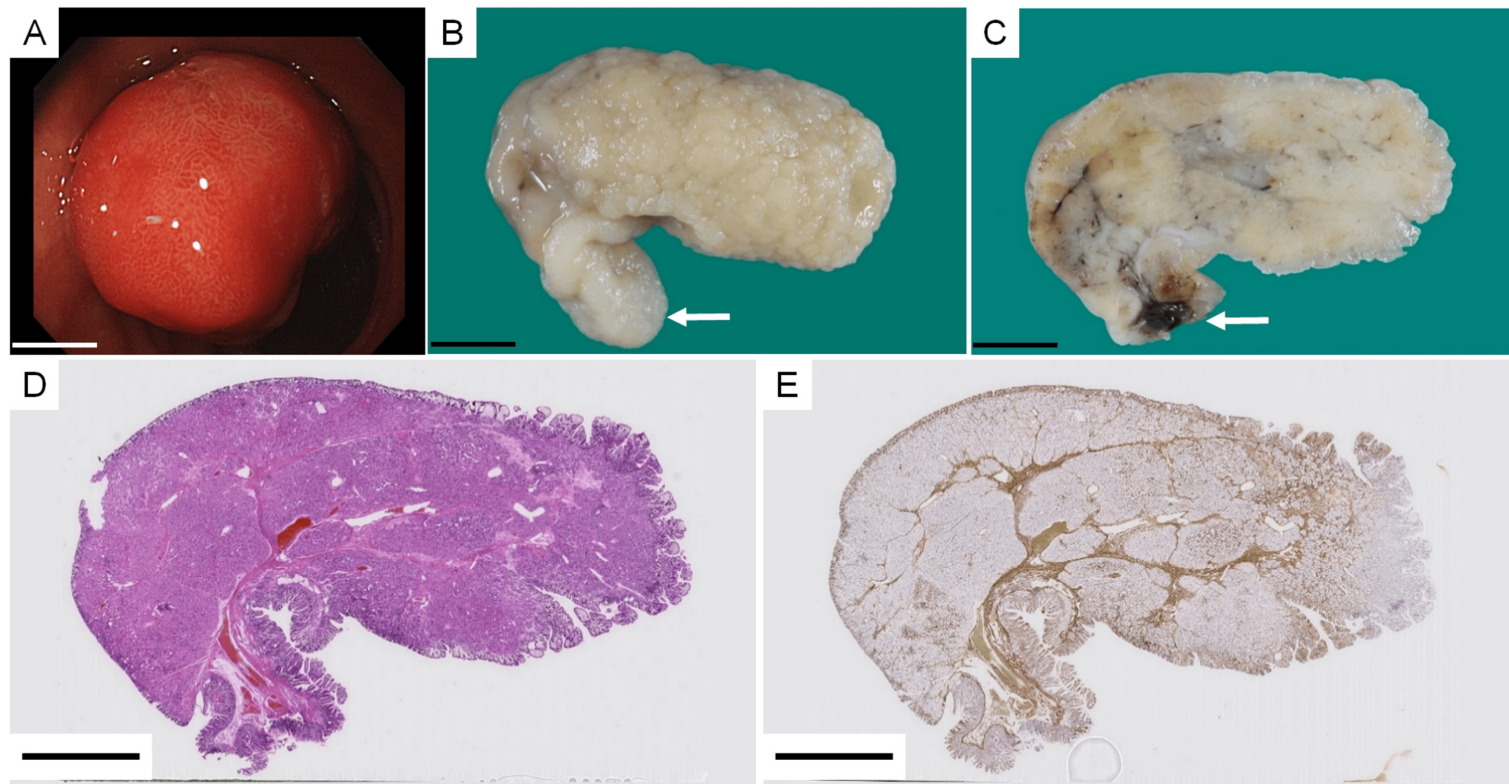
Gross specimen and loupe images of the duodenal polyp (A) Endoscopic view of a pedunculated polyp in the duodenal bulb. (B) Formalin-fixed, pedunculated polyp with a yellow-white appearance (40 mm × 34 mm × 24 mm). The white arrow indicates the polypectomy resection margin. (C) Cross-section of the formalin-fixed polyp. The white arrow indicates the polypectomy resection margin. (D) The loupe image of the polyp stained with hematoxylin-eosin (HE). (E) The loupe image of the polyp immunostained with anti-smooth muscle actin (SMA) antibody. The scale bars represent 10 mm.

The patient was a 57-year-old Japanese man who had been under medical care for a history of epilepsy since his teenage years. The patient had no gastrointestinal symptoms; however, an upper gastrointestinal endoscopy was performed during a health checkup. Diffuse atrophy of the gastric mucosa was observed throughout the entire stomach. Additionally, five to six sessile polyps, approximately 5 mm in diameter, were detected from the gastric fundus to the body. A 15-mm pedunculated polyp was observed on the greater curvature of the gastric angle. All polyps were endoscopically diagnosed as hyperplastic polyps. In particular, the 15-mm polyp was removed via endoscopic polypectomy. It was histologically confirmed as a hyperplastic foveolar polyp. A pedunculated polyp was also discovered in the duodenal bulb (Figure 1A), for which endoscopic polypectomy was performed. The polyp was pedunculated, yellow-white, and measured 40 mm × 34 mm × 24 mm (Figures 1B, 1C). Under loupe magnification, the duodenal polyp exhibited a lobulated structure with the fibromuscular connective tissue (Figures 1D, 1E).

**Figure 2:**
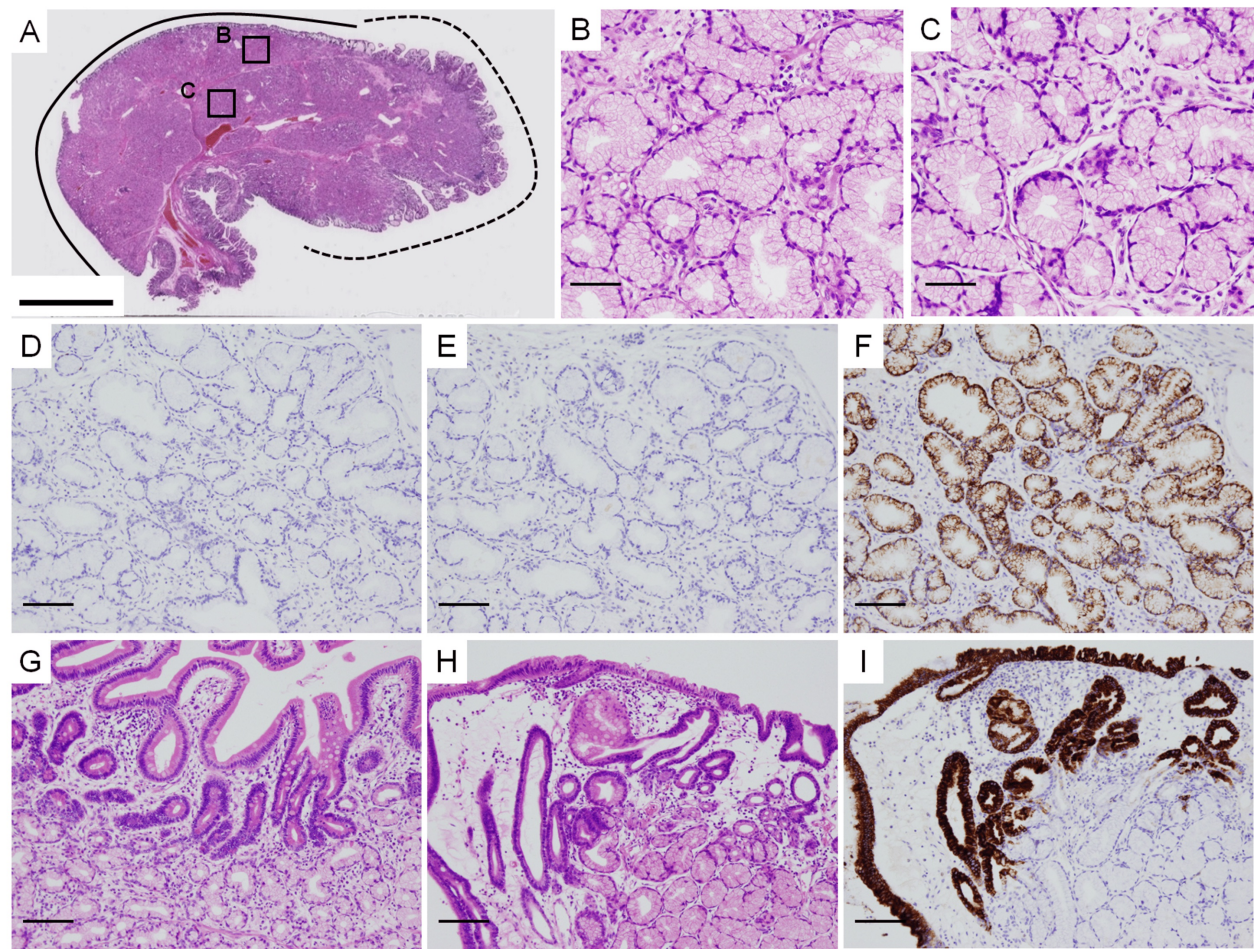
Brunner's gland hyperplasia (A) Loupe image of the polyp stained with HE; rectangles B and C correspond to the areas shown in (B) and (C), respectively. (B) HE staining of Brunner’s glands without atypia in the superficial part of the polyp (the presumed mucosal layer) (×200). (C) HE staining of Brunner’s glands without atypia in the deep part of the polyp (the presumed submucosal layer) (×200). Immunostaining with anti-Ki-67 antibody (D), anti-mucin 5AC antibody (E and I), and anti-mucin 6 antibody (F) (×100). HE staining images of intestinal epithelium (G) and gastric foveolar metaplasia (H) as the covering epithelium (magnification, ×100). The covering epithelium on the right side of the polyp (dashed line in A) consists of intestinal epithelium (G), whereas on the left side of the polyp (solid line in A), it is composed of gastric foveolar metaplasia (H). The scale bars represent 10 mm (A), 50 µm (B and C), and 100 µm (D-I).

The proliferating glandular epithelium had no atypia, nuclear enlargement, nucleoli, or mitotic figures (Figures 2A-2C). Immunohistochemical examination revealed that the Ki-67 labeling index was extremely low overall (Figure 2D), mucin 5AC staining was negative (Figure 2E), and mucin 6 staining was positive (Figure 2F), which is consistent with Brunner’s gland mucin. The covering epithelium consisted of intestinal epithelium (Figure 2G) on the right side of the polyp (the dotted line in Figure 2A) and gastric foveolar metaplasia (Figures 2H, 2I) on the left side (the solid line in Figure 2A). Differential diagnoses included Brunner’s gland hyperplasia, Brunner’s gland hamartoma, and Brunner’s gland adenoma (i.e., pyloric gland adenoma). No findings indicated Brunner’s gland hamartoma such as mature adipocytes or large ducts. Additionally, no nuclear enlargement or prominent nucleoli and no ectopic gastric fundic glands were detected in the tissue. Therefore, this polyp was not considered a Brunner’s gland adenoma (i.e., pyloric gland adenoma). This polyp was ultimately diagnosed as Brunner’s gland hyperplasia.

**Figure 3:**
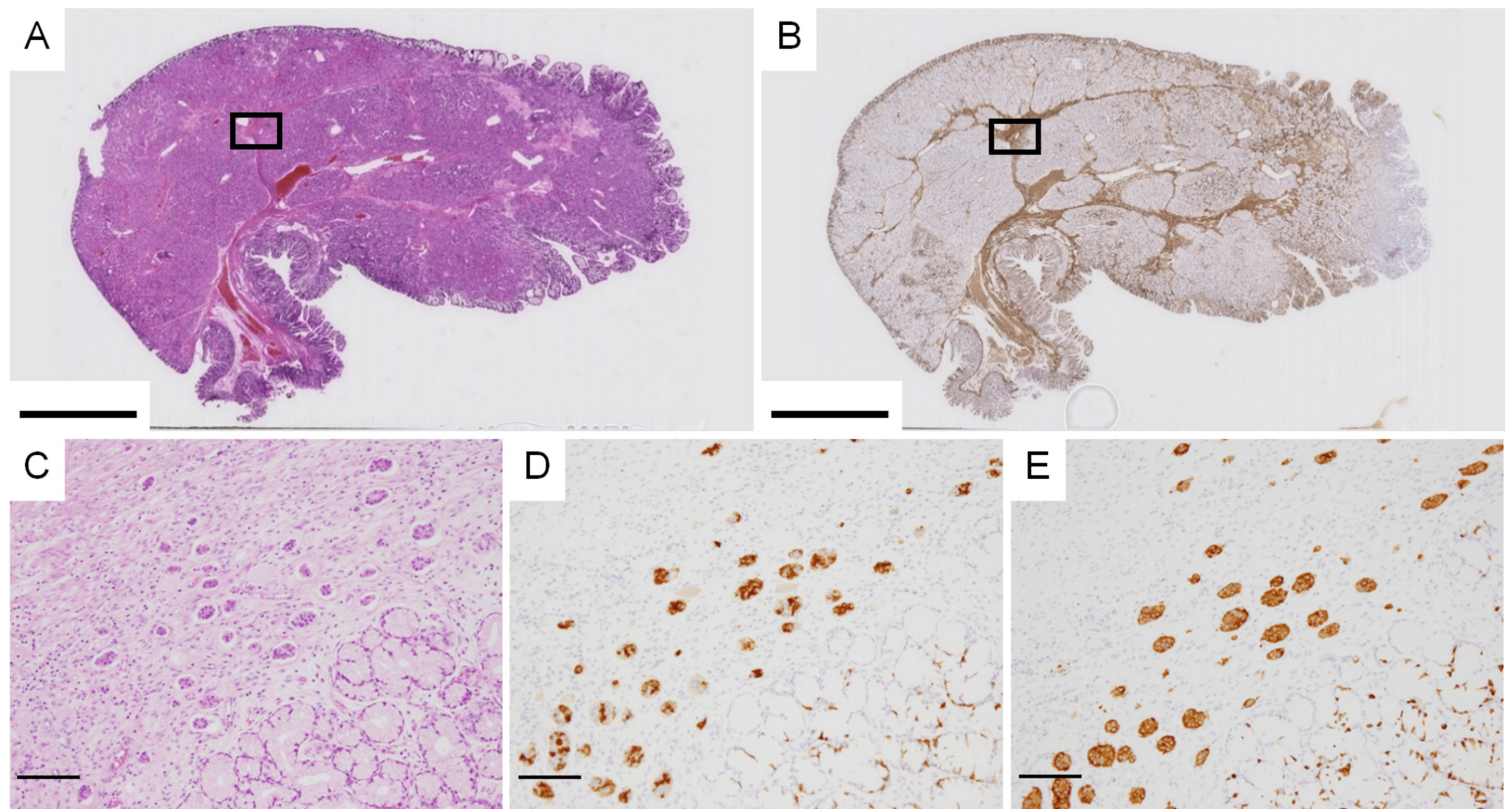
Brunner’s gland hyperplasia and endocrine cell micronests (A) The loupe image of the polyp stained with HE. (B) The loupe image of the polyp immunostained with anti-SMA antibody. Rectangles in (A) and (B) correspond to (C). (C) Small clusters of cells in the SMA-positive fibromuscular connective tissue (magnification, ×100). Immunostaining with anti-chromogranin A antibody (D) (×100) and anti-synaptophysin antibody (E) (×100) reveals that the small cell clusters are composed of endocrine cells positive for chromogranin A and synaptophysin, respectively. The scale bars represent 10 mm (A and B) and 100 µm (C-E).

Additionally, small nests of cells measuring approximately 50 µm in diameter were found within the fibromuscular connective tissue (Figures 3A, 3B). These cell clusters were identified as ECMs and were positive for chromogranin A (Figure 3C) and synaptophysin (Figure 3D).

**Figure 4:**
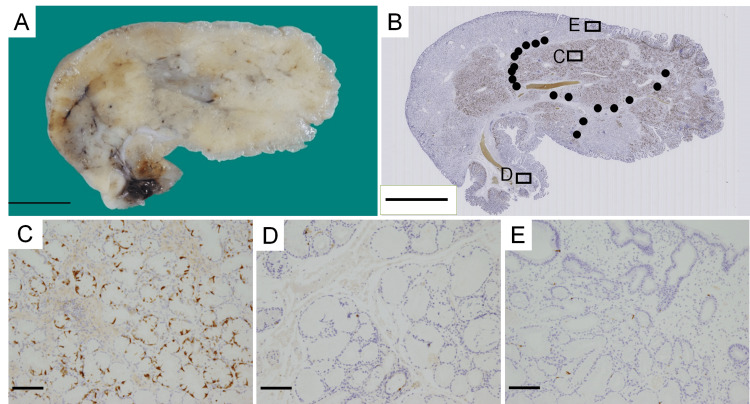
Distribution of endocrine cells and endocrine cell micronests (ECMs) in hyperplastic Brunner’s glands (A) The cut surface of the formalin-fixed polyp. (B) The polyp is immunostained with anti-chromogranin A antibody. The black circles correspond to approximately 10 ECMs and rectangles C, D, and E correspond to (C), (D), and (E), respectively. (C) Deep Brunner’s glands immunostained with anti-chromogranin A antibody (magnification, ×100). (D) Normal Brunner’s glands near the resection margin immunostained with anti-chromogranin A antibody (×100). (E) Superficial Brunner’s glands immunostained with anti-chromogranin A antibody (×100). The scale bars represent 10 mm (A and B) and 100 µm (C-E).

In addition, we noticed that endocrine cells in hyperplastic Brunner’s glands were more abundant in the deeper regions (presumed as the submucosa) (Figures 4A, 4B). The number of endocrine cells in the deeper hyperplastic Brunner’s glands (Figure 4C) was higher than that in the normal Brunner’s glands near the resection (Figure 4D) and the superficial hyperplastic Brunner’s glands (Figure 4E). ECMs were absent in hyperplastic Brunner’s glands with fewer endocrine cells but existed only in the fibromuscular connective tissue of hyperplastic Brunner’s glands with more endocrine cells (Figure 4B). However, compared with normal Brunner’s glands, hyperplastic Brunner’s glands did not have increased glandular density or cellular atypia, regardless of the presence or absence of endocrine cells.

**Figure 5:**
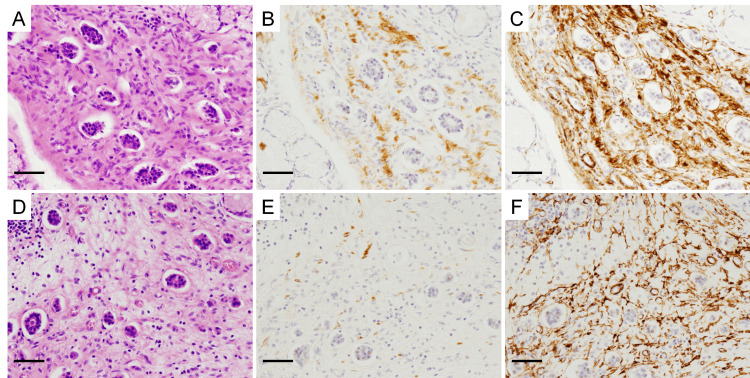
Smooth muscle cells and myofibroblasts coexist around endocrine cell micronests (ECMs) (A and D) HE staining of the fibromuscular connective tissue (magnification, ×200). (B and E) Fibromuscular connective tissue containing ECMs, immunostained with anti-desmin antibody (×200). (C and F) Fibromuscular connective tissue containing ECMs, immunostained with anti-SMA antibody (×200). The scale bars represent 50 µm.

When the cells surrounding the ECMs were examined via immunohistochemical staining, the ECMs (Figure 5A) were surrounded by a mixture of desmin-positive smooth muscle cells (Figure 5B) and desmin-negative/alpha-smooth muscle actin-positive myofibroblasts (Figure 5C). In another ECM (Figure 5D), a mixture of smooth muscle cells (Figure 5E) and myofibroblasts (Figure 5F) was observed, with myofibroblasts being predominant. In Figure 5F, myofibroblasts appeared to be located close to the ECMs. Around all observed ECMs, smooth muscle cells and myofibroblasts were noted, although their proportions varied.

**Figure 6:**
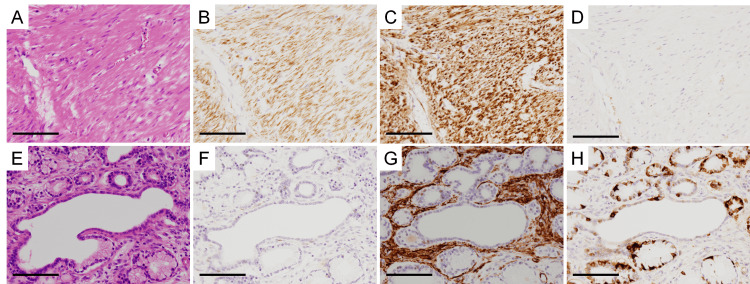
Endocrine cell micronests (ECMs) are not observed in smooth muscle cell layers with few myofibroblasts or in fibrotic areas with few smooth muscle cells (A-D) Smooth muscle cell layer without myofibroblasts (magnification, ×100). (A) HE staining. (B) Immunostaining with anti-desmin antibody. (C) Immunostaining the anti-SMA antibody. (D) Immunostaining with anti-chromogranin A antibody. (E-H) Fibrotic areas with few smooth muscle cells (×100). (E) HE staining. (F) Immunostaining with anti-desmin antibody. (G) Immunostaining with anti-SMA antibody. (H) Immunostaining with anti-chromogranin A antibody. The scale bars represent 200 µm.

By contrast, no ECMs were observed in the smooth muscle cell layer where myofibroblasts were absent (Figures 6A-6D) or in fibrotic areas within the endocrine cell-rich hyperplastic Brunner’s glands where smooth muscle cells were absent (Figures 6E-6H).

Larger ECMs with diameters exceeding 100 μm were not observed within the duodenal polyp, and no carcinoids were detected. The gastric polyps were identified as foveolar-type hyperplastic polyps. However, the sampled tissue did not include the nonhyperplastic gastric mucosa. Thus, atrophy of the gastric fundic glands and ECMs was not confirmed. No biopsies were taken from the gastric mucosa, which appeared atrophic based on endoscopic findings.

Following the polypectomy, serum gastrin and serum vitamin B12 levels were measured. The serum gastrin level was elevated to 2,500 pg/mL (reference range: 30-140 pg/mL), and the serum vitamin B12 level was decreased to 61 pg/mL (reference range: 180-914 pg/mL). However, anti-parietal cell and anti-intrinsic factor antibodies were both negative. Anti-*Helicobacter pylori *antibodies were positive (62 U/mL, reference range: <10 U/mL). A whole-body CT scan did not reveal any tumorous lesions suggestive of gastrinoma in the pancreas or other organs. Owing to circumstances, the patient was transferred to another hospital, and a biopsy to confirm ECMs in the stomach was not performed at our hospital. Autoimmune gastritis consequently was not histologically confirmed.

## Discussion

In this paper, we reported a case of Brunner’s gland hyperplasia in the duodenal bulb with increased endocrine cells and the presence of ECMs. In this patient, hypergastrinemia may have induced the formation of ECMs. ECMs are very rarely observed in gastric lesions, other than gastric carcinoids, although they have been reported. Anjiki et al. [2] reported a case of hypergastrinemia (serum gastrin level: 10,206 pg/mL (reference range: 37-172 pg/mL)) and ECMs in a patient with adenocarcinoma within a hyperplastic polyp detected during long-term proton pump inhibitor therapy for *H. pylori* infection. Additionally, Yoshida et al. [3] reported three cases of ECMs in adenocarcinomas arising from autoimmune gastritis, with serum gastrin levels of 1,954 pg/mL, 6,333 pg/mL, and 2,848 pg/mL (reference range: 5-150 pg/mL), respectively. These findings suggested that the increase in serum gastrin induces the production of gastrin by gastric cancer epithelial cells expressing cholecystokinin-B receptors, which then stimulates the growth and metastasis of gastric cancer via an autocrine mechanism [8].

Based on a study by Sheng et al. [9] demonstrating that gastrin increases the number of enterochromaffin-like cells, the increase in endocrine cells in the hyperplastic Brunner’s glands was attributed to hypergastrinemia. However, compared with normal Brunner's glands, hyperplastic Brunner’s glands showed no apparent increase in glandular density or cellular atypia, regardless of the presence or absence of endocrine cells. Although the mechanism is unclear, a possibility is that gastrin increases the proportion of endocrine cells within the glandular ducts. ECMs were only observed in regions with hyperplastic Brunner’s glands rich in endocrine cells but not in regions with normal endocrine cell numbers, suggesting a correlation between increased endocrine cells and ECM formation. Itsuno et al. [1] hypothesized that hyperplastic and clustered endocrine cells bud from gastric glands into the extraglandular space under hypergastrinemia. However, this process has not been proven, and the mechanism of ECM formation remains unclear. This case had no morphological evidence of endocrine cells budding from glandular ducts into the extraglandular space.

In this case, all ECMs were surrounded by a mixture of smooth muscle cells and myofibroblasts. However, no ECMs were in the smooth muscle cell layer, which may correspond to the muscularis mucosae, where myofibroblasts were mostly absent, or in the fibrotic areas where smooth muscle cells were absent. These findings suggested that smooth muscle cells and myofibroblasts, which are presumably derived from smooth muscle cells, have a crucial role in forming the ECM microenvironment. However, the presence of myofibroblasts around ECMs could also result from chronic inflammation, which may cause their incidental formation around ECMs. Further investigation of ECM cases is necessary to determine whether the presence of myofibroblasts around ECMs is a common phenomenon or whether it is specific to this patient. The significance of myofibroblasts around ECMs in this case should be carefully considered.

Causes of hypergastrinemia include acid-suppressing drugs, *H. pylori*-associated atrophic gastritis, autoimmune gastritis, and gastrinoma [10]. The patient had no history of acid-suppressing medication use, had no anti-*H. pylori* antibodies, and had no evidence of gastrinoma, based on a whole-body CT scan. This finding suggested possible autoimmune gastritis.

The diagnosis of autoimmune gastritis typically involves endoscopic findings (e.g., atrophy of the gastric body mucosa), histopathological findings, autoantibodies against parietal cells and intrinsic factors, and serum gastrin levels [11]. In this patient, autoantibodies against parietal cells and intrinsic factor were negative, despite having hypergastrinemia (2,500 pg/mL), endoscopic findings of atrophic gastritis, and low vitamin B12 levels. The gastric mucosa biopsy did not confirm the atrophy of the fundic glands or ECMs; therefore, a definitive diagnosis was not reached. However, the autoimmune gastritis cannot be completely ruled out.

The limitations of this case report included the insufficient histopathological evaluation of autoimmune gastritis and the inability to explain why the number of endocrine cells did not increase in the hyperplastic Brunner’s glands occupying the superficial region.

## Conclusions

We reported a case of Brunner’s gland hyperplasia with increased endocrine cells and ECMs. To the best of our knowledge, this paper is the first reported instance of Brunner’s gland hyperplasia associated with ECMs. This case may help elucidate the relationship among hyperplasia of Brunner’s gland endocrine cells, ECMs, and hypergastrinemia. However, more similar cases are needed to fully understand this relationship.

## References

[1] Itsuno M, Watanabe H, Iwafuchi M (1989). Multiple carcinoids and endocrine cell micronests in type A gastritis. Their morphology, histogenesis, and natural history. Cancer.

[2] Anjiki H, Mukaisho KI, Kadomoto Y (2017). Adenocarcinoma arising in multiple hyperplastic polyps in a patient with Helicobacter pylori infection and hypergastrinemia during long-term proton pump inhibitor therapy. Clin J Gastroenterol.

[3] Yoshida K, Yamatsuji T, Matsubara M (2019). Four cases of gastric cancer in patients with autoimmune gastritis. Kawasaki Med J.

[4] Abe H, Kubota K, Oka T, Kobayashi T, Makuuchi M (2000). A rare case of multiple carcinoids and endocrine cell micronests in a patient with chronic duodenitis. Cancer.

[5] Noda Y, Watanabe H, Iwafuchi M (1992). Carcinoids and endocrine cell micronests of the minor and major duodenal papillae. Their incidence and characteristics. Cancer.

[6] Suda K (2010). Histopathology of the minor duodenal papilla. Dig Surg.

[7] Terada T (2012). Pathologic observations of the duodenum in 615 consecutive duodenal specimens: I. benign lesions. Int J Clin Exp Pathol.

[8] Smith JP, Nadella S, Osborne N (2017). Gastrin and gastric cancer. Cell Mol Gastroenterol Hepatol.

[9] Sheng W, Malagola E, Nienhüser H (2020). Hypergastrinemia expands gastric ECL cells through CCK2R(+) progenitor cells via ERK activation. Cell Mol Gastroenterol Hepatol.

[10] Dacha S, Razvi M, Massaad J, Cai Q, Wehbi M (2015). Hypergastrinemia. Gastroenterol Rep.

[11] Kamada T, Maruyama Y, Monobe Y, Haruma K (2022). Endoscopic features and clinical importance of autoimmune gastritis. Dig Endosc.

